# Irrational beliefs indirectly predict retirement satisfaction through the conceptualization of retirement: a cross-sectional study in a sample of recent retirees

**DOI:** 10.1186/s40359-023-01237-9

**Published:** 2023-07-04

**Authors:** Viera Bačová, Peter Halama, Jana Kordačová

**Affiliations:** Institute of Experimental Psychology, Centre of Social and Psychological Sciences, Slovak Academy of Sciences, Dúbravská Cesta 9, 841 04 Bratislava, Slovak Republic

**Keywords:** Retirement satisfaction, Retirement concepts, General irrational beliefs, Retirement transition

## Abstract

**Background:**

Although most retirees are satisfied, some do not feel well in retirement. The resource-based dynamic perspective explains retirement dissatisfaction as the lack of resources. This study focused on psychological resources, specifically on the role of rational/irrational beliefs and retirement concepts in retirement satisfaction. While irrational beliefs have many consequences, we know little about their role in retirement experiences, nor do we know about the benefits/harm of retirement concepts for retirement satisfaction. We assumed that not succumbing to irrational beliefs and conceptualizing retirement actively and positively add to psychological resources helping to adjust to retirement and retirement satisfaction. Our objective was to examine whether irrational beliefs and retirement concepts contributed to satisfaction or dissatisfaction in recent retirees.

**Methods:**

200 recent retirees (average retirement time 2.8 years) completed questionnaires containing the Irrational Belief Scale, the Satisfaction with Retirement Scale, and the Retirement Lifestyles Questionnaire, which determines the inclination toward four retirement concepts: Transition to Old Age, New Start, Continuation, and Imposed Disruption. The Pearson correlation coefficients were used to estimate the relationship between irrational beliefs, retirement concepts, and retirement satisfaction. We used a parallel mediation model with multiple mediators in the mediation analysis where irrational beliefs were the independent variable, retirement satisfaction was the dependent variable, and the four retirement concepts were mediators.

**Results:**

We confirmed higher retirement satisfaction in recent retirees who conceptualize retirement as a *New Start* and *Continuation* and higher retirement dissatisfaction in those who see retirement as an *Imposed Disruption* or *Transition to Old Age*. The general irrational beliefs had a weaker direct impact on retirement satisfaction than the more specific retirement concepts. Inclination to general irrational beliefs appeared only weakly reflected in retirement dissatisfaction. However, a negative view of retirement as an imposed disruption might increase this inclination by intensifying retirement dissatisfaction.

**Conclusions:**

Our results show a negative retirement concept as an imposed disruptive event that amplifies the impact of general irrational beliefs and leads to retirement dissatisfaction in recent retirees. It suggests that using rational-emotive behavior therapy and interventions to change the negative perception of retirement could be effective in increasing retirement satisfaction.

## Background

### Impact of retirement on satisfaction

There is a wealth of studies on the implications of retirement for individual well-being and satisfaction of retirees. In recent empirical studies, researchers have concluded that retirement, in general, is positively associated with well-being. Most retirees maintain their level of well-being over retirement and, recently, robust evidence has been presented showing that retirement causally improves overall life satisfaction, finding more improvements associated with retirement in terms of satisfaction with one's financial situation, free time, health, and participation in local community activities [1]. Also, after considering sociodemographic and health indicators, life satisfaction was higher for retired than for non-retired individuals [2]. Retirement improved physical as well as mental health, and life satisfaction [3]. Large-scale research studies also specified more detailed conditions for improving satisfaction in retirement. Life satisfaction significantly increased for a great majority of individuals who retired voluntarily from the labor force [4]. Moreover, in people retiring from employment, life satisfaction did not change in the short term. In the long term, however, satisfaction became more positive [5]. Similarly, Sohier, Van Ootegem, and Verhofstadt conclude their research by stating that “on average, people do not immediately report a different level of life satisfaction when retiring, but in social-democratic countries, there is a positive effect” [p. 279, [6]]. This effect was confirmed in a cross-cultural study in sixteen countries [7].

Regardless of the findings revealing that most retirees are satisfied in retirement, smaller groups remain who suffer from retirement for various reasons [8]. Since retirees are not a homogeneous group, individuals vary in adjustment to this phase of life. The diverse outcomes of retirement have been documented and some studies have provided evidence for substantial heterogeneity in change and continuity in well-being during the retirement transition and in retirement [9–11]. Wang [9] and Pinquart and Schindler [10] independently found similar patterns using indicators of well-being, life satisfaction, and leisure satisfaction [see also [12, 11]]. They identified three subgroups: participants who increase, decrease or maintain their scores in retirement satisfaction or well-being after transitioning into retirement. The impact of retirement also varies over time for individuals. Wetzel, Huxhold, and Tesch-Romer [13] and Schmälzle et al. [5] indicated that some changes affect retirees in the short term (change in social status), others require long-term adjustment (changes in the resources of individuals; resources were measured primarily by education).

### Psychological resources for satisfaction in retirement

Wang with his colleagues proposed a dynamic, resource-based model [9, 14] which helps explain heterogeneity in retirement responses. The central premise of the model is that retirement adjustment depends directly on whether individuals have the resources at their disposal [14]. If people have more resources available, they will experience less difficulty in adjusting to retirement adjustment and in retirement life in general. On the other hand, the lack of or decline in resources hurts retirement adjustment and satisfaction. Wang [9] identified six types of such resources, namely physical, financial, social, cognitive, motivational, and emotional ones. For the most part, these resources are measured as participants perceive them (self-rated physical health, financial satisfaction, perceived social support) [see also [15]]. Hansson et al. [16] argue that perceived resource adequacy is more important for life satisfaction than objective circumstances [17]. Psychological resources are studied the least [18], even though they significantly affect how individuals react to and cope with changes associated with retirement life [8]. Additional authors also note a lack of in-depth studies on the role of psychological resources in explaining the heterogeneity in well-being and satisfaction in retirement ([8, 19]. Further, the operationalization of psychological resources has varied in different research studies. Perceived personal control, self-esteem, autonomy, and self-rated cognitive ability have been used most frequently [19–21]. To the best of our knowledge, an association between personal beliefs and retirement satisfaction has not been examined as of yet, thus, our research seeks to expand knowledge about the implications of rational/irrational beliefs and retirement concepts on satisfaction with retirement.

### Irrational/rational personal beliefs as psychological resources of life dis/satisfaction

Personal beliefs, according to our understanding, are views of how the world functions and how we as individuals fit into this world, including retirement concepts that individuals develop about retirement. We consider personal beliefs part of the psychological resources or barriers that contribute to life management in a positive or negative direction, depending on the nature of such beliefs. Concerning satisfaction in retirement, we are dealing with the role of general or specific personal beliefs, as well as rationality/irrationality and positivity/negativity of personal beliefs.

The concept of irrational/rational beliefs was developed within Ellis's rational-emotive behavior therapy (REBT) model. The model highlights the integral role of cognition in adaptive and maladaptive functioning [22, 23]. According to the model, individuals have general rational (adaptive, healthy, or functional) or irrational (maladaptive, unhealthy, or dysfunctional) beliefs activated by various upcoming events. The beliefs have emotional, behavioral, and cognitive consequences in the functioning of a person. In the life of an individual, rational beliefs consistent with reality are constructive, whereas irrational beliefs inconsistent with reality are detrimental [22]. Beliefs are formed in the time between the actual event and its consequences and thus mediate the way people perceive the event [23].

General rational/irrational beliefs are considered cross-situational, aggregated, superordinate, decontextualized, and overgeneralized [24]. Accordingly, almost all scales measuring rational/irrational beliefs use abstract and generalized items (such as “Everybody must respect me”). However, some scales of rational/irrational beliefs in specific domains have been constructed (for example, ones about parenting aimed at parents [25] or employment targeting the adult working population [26]). As far as we know, there is no measurement tool to assess rational/irrational beliefs about retirement. However, specific instruments used to assess retirement [27] can reveal retirement beliefs, when they contain personal experiences about the world and oneself related to retirement. According to the REBT model, the inclination to rational beliefs is favorable for the positive functioning of the individual in all areas of life.

Concerning life satisfaction, several studies found elevated dysfunctional/irrational beliefs associated with lower life satisfaction, either directly or through intervening variables. Snell and Hawkins [28] found that irrational beliefs diminish people's satisfaction with life, especially with recently experienced negative life changes. Ciarrochi [29] showed dysfunctional beliefs as being related substantially to negative indicators and moderately to positive indicators of well-being and life satisfaction. In their research, Froh et al. [30] indicated that interpersonal relations mediated the association between global irrationality and life satisfaction, while Balkıs and Duru [31] revealed that irrational beliefs indirectly predict life satisfaction via procrastination. Furthermore, Strobel, Bekk, and Spörrle [32] uncovered medium-sized negative correlations between irrational beliefs and life satisfaction unaffected by age, sex, and social desirability. In the second study, Spörrle, Strobel, and Tumasjan [33] revealed a significant effect of irrationality over personality factors in predicting life satisfaction but not happiness.

### General personal beliefs versus specific retirement beliefs

It remains unclear whether and how general irrational beliefs affect global life satisfaction and retirement satisfaction throughout life. However, referring to previous findings, we can assume that not succumbing to the feeling of helplessness, idealization, perfectionism, vulnerability, and negative expectations (which are the dimensions of irrational beliefs in our study) helps increase satisfaction with retirement life and thereby with life in general.

One of the differences between general and specific beliefs lies in their temporal location in individual development. According to the founders of Rational emotive behavior therapy, there is a congenital biological tendency toward irrational alongside rational thinking, which develops in the early psychosocial environment through to adulthood. Family thinking patterns are recognized as an essential source of general irrational beliefs [34]. General personal beliefs can arise at a very early age and can affect many aspects of personal life later on. The specific content of personal beliefs is formed dominantly in those domains that are significant for the given phase. It is usually only in later life when pre-retirees develop their ideas about retirement, elaborate on their retirement attitudes, and specify their retirement expectations and intentions [35]. Concerning the specificity of personal beliefs in retirement, we believe that general irrational/rational beliefs affect retirement satisfaction through retirement-specific personal beliefs.

### Retirement concepts as psychological resources of retirement satisfaction

Individuals can assess their retirement differently as they attribute different meanings to retirement and hold different personal beliefs about this phase of life. In terms of retirement experiences, Hornstein and Wapner [36] in their qualitative research study identified four conceptualizations in participants. In the first conceptualization, pre-retirees and retirees understood retirement as the *transition to old age*, to a phase of life where it is time to reduce activity, rest, achieve a balance, and put one's life "in order." In the second retirement concept, retirees welcomed retirement as the *beginning* of a whole new phase in life, as a time to begin living life according to one’s needs, desires, and goals, free from the demands of others. Other participants did not see retirement as a big event, since their current life did not differ much from the previous one. They *continued* their activities in a more satisfactory and less demanding way (the third concept). In the fourth conceptualization, retirement meant the loss of the world of work, the loss of a highly valued activity for which there is no compensation. As a result, the participants found retirement to be a frustrating period. These retirement concepts have been heavily used in subsequent research studies, in which authors developed questionnaires as tools to identify them [37, 38]. Some of the results suggest a combination of *New Start* and *Continuation* concepts with positive activities [39, 40] and the concept *of Transition to Old Age* with in-activity [41]. The *Imposed Disruption* concept binds to itself the most negative connotations and retirement outcomes [39, 41].

We consider the concepts of retirement an expression of retirement-specific beliefs. The relationships between general and specific retirement beliefs we specify as follows. General rational beliefs would positively link to positive retirement concepts and subsequently to retirement satisfaction. Negative retirement concepts would reinforce the adverse impact of general irrational beliefs on retirement satisfaction.

### The life/retirement satisfaction assessment

The successful adjustment to retirement has been long measured by life satisfaction [12, 14, 42]. Life satisfaction is the cognitive component of subjective well-being based on attitudes and beliefs about one's life [43]. By analogy, satisfaction with retirement is the cognitive component of a retiree's well-being based on attitudes and beliefs about one's life in retirement. Amorim and França [44] conceived satisfaction in retirement as a subjective sense of well-being, representing a sense of contentment during this phase of life. Referring to other authors, they state that unlike adjustment to retirement, satisfaction in retirement does not refer to a process but is rather an indicator of well-being and quality of current life in retirement. Similarly, Henning et al. [45] used three indicators of retirement adjustment. The first indicator focused on perceived change in life after retirement. The second item measured directly perceived retirement satisfaction, and the third question focused on adjustment difficulty. Since the global life satisfaction indicator does not capture retirement adjustment aptly, in this study we choose to measure satisfaction directly by the specific questions about life in retirement.

### The present study

Although irrational beliefs predict many consequences, including life dissatisfaction, we know little about their role in retirement experiences. We know just as little about the benefits/harm of retirement conceptualization for retirement satisfaction. This study expands knowledge of the implications of irrational beliefs and retirement concepts on retirees' life. Although we do not neglect objective socio-economic conditions and circumstances, we assume that not succumbing to irrational beliefs and conceptualizing retirement actively and positively will add to those psychological resources that help a person adjust to retirement and secure subsequent retirement satisfaction. Our objective is to examine whether irrational beliefs and retirement concepts contribute to satisfaction or dissatisfaction with retirement.

Based on previous findings and considerations, we hypothesized that active and positive retirement concepts such as a *New Start* and *Continuation* would relate positively to retirement satisfaction *(H1)*. The retirement concepts with a negative connotation, the *Transition to Old Age* and *Imposed Disruption,* would relate negatively to retirement satisfaction *(H2).*

Further, we assume that general irrational beliefs would correlate negatively with retirement satisfaction *(H3).* As for the relationship of general irrational beliefs to retirement concepts, we hypothesized that they would be associated negatively with positive retirement concepts *(H4)* and positively with negative retirement concepts *(H5).*

We also tested a mediation model and assumed that retirement concepts mediate the effect of general irrational beliefs on retirement satisfaction *(H6).* Although we are aware of the cross-sectional design of our research, we believe that theoretical assumptions are strong enough to formulate a mediation model with irrational beliefs as predicting (X) variables. As discussed earlier, general irrational beliefs conceptualized in our study (within Ellis's REBT model) come from past experiences, possibly as early as childhood. On the other hand, retirement concepts are emerging later when a person starts facing retirement issues, or sometimes even later, only in retirement. We, therefore, believe that the theoretical time framework justifies the mediation model with irrational beliefs as predictors and retirement concepts as mediators and that the model represents properly the nature of the psychological mechanism behind the effect of irrational beliefs on retirement satisfaction: the general irrational beliefs can have an effect on retirement concepts and subsequently increase or decrease retirement satisfaction.

In our study, we chose a parallel mediation approach [46]. The advantage of a parallel model over simple mediations is that parallel mediation enables controlling for the effects of other mediators on mediation effects; therefore, the regression coefficients reflect more accurately the unique contribution of the individual mediators. This leads to indirect effects being more accurate because they are purified from the effects of other mediators.

## Method

### Sample

The research sample consisted of 200 retirees, of which 101 were women. All participants were from Slovakia. The participants' ages varied from 60 to 69, with an average of 63.35 years. The average time since the onset of retirement was 2.8 years. One hundred thirty-four were married or in a romantic relationship, 39 were divorced, 19 were widowed, and eight were single. One hundred sixty-one lived with their family or other people, and 39 lived in a one-person household. One participant had a basic education, 122 had a high school education and 77 had a university degree. An online research agency recruited the participants. Participation in the study was voluntary and anonymous. All participants provided informed consent before participation.

### Measures

To measure irrational beliefs**,** we used the Irrational Belief Scale [47]. The scale measures inclination to irrational beliefs as defined in Ellis’s rational-emotive behavioral theory. It contains 40 items answered on a 5-point Likert-type scale (from *I am not convinced that´s true* to *I am convinced that´s true)* and forms five dimensions: Helplessness (10 items, e.g. *It is futile to defy fate and circumstance*), Idealization (8 items, e.g. *There should be no misunderstandings between people),* Perfectionism (8 items, e.g. *One should excel in all situations),* External Vulnerability (10 items, e.g. *Without the help of our loved ones, we would be completely lost*) and Negative Expectations (7 items, e.g. *Selfless people will always pay for the way they are in the end*). The higher number of items in dimensions comes from the fact, that several items belong to more than one dimension. The scale contains five supplementary questions intended for the qualitative analysis of participant thinking, which we do not use in the present study. The original authors [47] provided the following information on internal consistency in individual dimensions (Cronbach alpha): 0.77 for Helplessness, 0.67 for Idealization, 0.66 for Perfectionism, 0.63 for External Vulnerability, 0.60 for Negative Expectations, and 0.89 for all scales. Internal consistency for the current data was 0.82 for Helplessness, 0.65 for Idealization, 0.79 for Perfectionism, 0.72 for External Vulnerability, 0.67 for Negative Expectations, and 0.91 for all items on the scale.

To measure retirement concepts**,** we used the shortened version of the Retirement Lifestyles Questionnaire [39], inspired by Hopkins et al. [40] and Hornstein and Wapner [36]. This shortened version has 20 items measuring four retirements concepts: New Start (5 items, e.g. *Retirement is an opportunity for me to engage in activities other than the previous ones*), Continuation (5 items, e.g. *After retirement, I do almost everything I did before, only at a slower pace*), Imposed Disruption (5 items, e.g. *It is difficult for me to find something meaningful after retirement*), and Transition to Old Age (5 items, e.g. *With retirement, I'm becoming more aware of mortality*). The participants answered items on a 7-point Likert scale (from *completely disagree* to *completely agree*). The internal consistency of the original questionnaire (8 items per dimension) estimated by Cronbach alpha was 0.85 for New Start, 0.78 for Continuation, 0.87 for Imposed Disruption, and 0.65 for Transition to Old Age [39]. Internal consistency for the current data with shorter versions (5 items per dimension) was 0.80 for New Start, 0.65 for Continuation, 0.80 for Imposed Disruption, and 0.57 for Transition to Old Age.

Retirement satisfaction was measured using the 3-item *Satisfaction with Retirement Scale* inspired by van Solinge and Henkens [42]. The scale contained items that required a comparison of current retirement with expectations, pre-retirement periods, and overall retirement satisfaction. Participants answered items on a 3-point scale. The items were phrased**:** 1. I am enjoying my retirement *a/ better than expected, b/ as expected, and c/ not as much as I would have expected.* 2. Compared to the two years before my retirement, my life is *a/ better, b/ more or less the same, and c/ not as good.* 3. Overall, now in retirement, I am *a/ satisfied, b/ somewhere between, and c/ dissatisfied.* The retirement satisfaction score was computed as the sum of the answers to the items. A higher score means higher retirement satisfaction. Our participants’ scale showed an internal consistency of α = 0.82.

### Statistical analysis

**Fig. 1:**
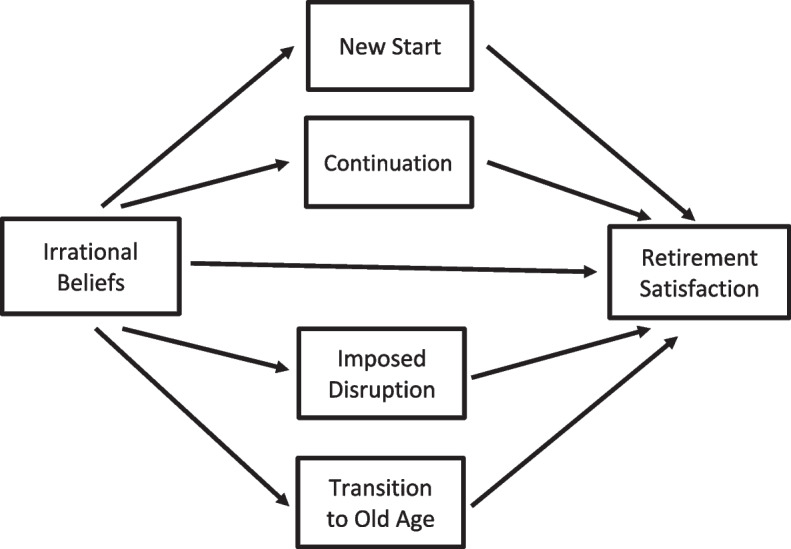
Parallel mediation model with Irrational Beliefs as the independent variable, Retirement Satisfaction as the dependent variable, and Retirements Concepts as parallel mediators

We performed all statistical analyses using IBM SPSS Statistics v. 27. The Pearson correlation coefficients were computed to estimate the relationship between irrational beliefs, retirement concepts, and retirement satisfaction. We used a parallel mediation model with multiple mediators in the mediation analysis [46]. To reduce the number of mediation models, we used the overall score of the Irrational Belief Scale as the predictor. Irrational beliefs were the independent variable, retirement satisfaction was the dependent variable, and retirement concepts were parallel mediators. Figure 1 displays the conceptual mediation model. The model was evaluated by testing the indirect effect based on linear regression using SPSS macro Process 3.2. [46]. The confidence interval of regression coefficients and effects was computed using the bootstrap method with 10 000 samples.

The data file with codes is publicly available at https://osf.io/hrf5a/?view_only=c7eb4a604ff74d3c92d22d62569f2d32

## Results

### Correlation analysis

As the first step, we computed descriptive characteristics of all variables used in the analysis (see Table 1). As seen from the skewness and kurtosis coefficients, none of the variables was heavily skewed or showed serious kurtosis (all coefficients are in a range from -1 to 1). Then, we computed the correlation coefficients between irrational beliefs, retirement concepts, and retirement satisfaction (Table 2). The retirement concepts correlated significantly with retirement satisfaction. As for *H1*, *New Start* and *Continuation* showed a moderate, positive correlation with Retirement Satisfaction (0.47 and 0.30 respectively). For *H2*, *Imposed Disruption* and *Transition to Old Age* showed a strong, negative correlation with Retirement Satisfaction (-0.61) respectively a weak, negative correlation (-0.21). The significant correlations between Irrational Beliefs and Retirement Satisfaction were weak and negative, ranging from -0.11 to -0.27 *(H3)*. Irrational beliefs positively correlated with the concepts of *Imposed Disruption* and *Transition to Old Age (H5)*, but not with *New Start* and *Continuation (H4)*. The significant correlations were weak to moderate and positive (from 0.19 to 0.42).

**Table 1:** Descriptive data for all variables

	Range	M	SD	Skewness	Kurtosis
*General Irrational Beliefs*					
1. Helplessness	13–49	29.18	6.59	0.19	-0.4
2. Idealization	15–39	28.60	4.09	-0.42	0.38
3. Perfectionism	12–37	23.82	5.03	0.06	-0.62
4. External Vulnerability	16–45	32.77	5.24	-0.30	0.11
5. Negative Expectations	11–32	20.89	4.25	0.02	-0.29
6. Irrational Beliefs—overall	77–183	126.15	18.84	0.13	-0.08
*Retirement Concepts*					
7. New Start	5–35	25.68	5.96	-0.70	0.75
8. Continuation	5–35	24.07	5.17	-0.22	0.07
9. Imposed Disruption	5–35	15.99	7.27	0.45	-0.52
10. Transition to Old Age	5–35	21.61	5.44	-0.12	-0.33
*Satisfaction with Retirement Life*					
11. Retirement Satisfaction	3–9	6.75	1.74	-0.51	-0.79

**Table 2:** Pearson correlations between Irrational Beliefs, Retirement Concepts, and Retirement Satisfaction

	1	2	3	4	5	6	7	8	9	10
1. Helplessness	–-									
2. Idealization	.46**	–-								
3. Perfectionism	.63**	.57**	–-							
4. External Vulnerability	.63**	.57**	.60**	–-						
5. Negative Expectations	.69**	.43**	.58**	.55**	–-					
6. Irrational Beliefs—overall	.86**	.73**	.84**	.82**	.79**	–-				
7. New Start	-.06	.05	-.03	-.03	-.14*	-.04	–-			
8. Continuation	.09	.07	.05	.04	-.01	.06	.32**	–-		
9. Imposed Disruption	.28**	.19**	.25**	.31**	.36**	.33**	-.42**	-.24**	–-	
10. Transition to Old Age	.41**	.25**	.28**	.35**	.42**	.42**	-.02	-.10	.43**	–-
11. Retirement Satisfaction	-.15*	-.15*	-.17*	-.11	-.27**	-.20**	.47**	.30**	-.61**	-.21**

^*^ *p* <  = .05; ** *p* <  = .01

### The mediation model (H6)

In the next step, we estimated the mediation model. The results of the estimation are in Tables 3 and 4.

**Table 3:** Effects in the mediation model

Path	*β*	B	SE(B)	95% CI	
				LL	UL
IBs → RS (Total effect)	-.201	-.019	.006	-.032	-.006
IBs → RS (Direct effect)	-.051	-.005	.006	-.016	.006
IBs → Imposed Disruption	.330	.127	.026	.076	.178
IBs → Transition to Old Age	.421	,122	.019	.085	.152
IBs → New Start	-.044	-.014	.023	-.058	.030
IBs → Continuation	.059	.016	.020	-.022	.054
New Start → RS	.227	.066	.020	.030	.103
Continuation → RS	.115	.039	.020	.000	.070
Imposed Disruption → RS	-.482	-.115	.017	-.148	-.083
Transition to Old Age → RS	.029	.009	.021	-.031	.050

*Note. CI* Confidence, *LL* Lower limit, *UL* Upper limit, *IBs* Irrational Beliefs, *RS* Retirement Satisfaction

**Table 4:** Standardized indirect effects of Irrational Beliefs on Retirement Satisfaction via Retirement Concepts

Indirect effects	Standardized Effect	SE	95% CI	
			LL	UL
Total indirect effect	-.150	.051	-.251	-.049
IBs → New Start → RS	-.010	.017	-.046	.024
IBs → Continuation → RS	.007	.009	-.010	.028
IBs → Transition to Old Age → RS	.012	.027	-.043	.065
IBs → Imposed Disruption → RS	-.159	.039	-.240	-.087

*Note. CI* Confidence, *LL* Lower limit, *UL* Upper limit, *IBs* Irrational Beliefs, *RS* Retirement Satisfaction

After controlling for all mediating variables, the significant and negative total effect of the overall irrational beliefs score on retirement satisfaction diminished to non-significant and close-to-zero (direct effect), as Table 3 shows.

Irrational beliefs significantly and positively predicted concepts of *Imposed Disruption* and *Transition to the Old Age*. After controlling for irrational beliefs and all retirement concepts, two retirement concepts showed a significant effect on retirement satisfaction. The concept *New Start* showed positive prediction and *Imposed Disruption* showed a negative prediction of retirement satisfaction.

Table 4 presents bootstrapping indirect effects. The results show that the total indirect effect of irrational beliefs on retirement satisfaction via the retirement concept is statistically significant and negative in value (beta = -0.150, 95% CI (-0.251, -0.049)). However, a close look at the indirect effects for the individual mediators showed that there is only one retirement concept, which accounted for this total indirect effect and mediated the relationship between irrational beliefs and retirement satisfaction: *Imposed Disruption.* The mediating effect of *Imposed Disruption* was negative, which means that irrational beliefs increase the level of *Imposed Disruption* and *Imposed Disruption* decreases the level of retirement satisfaction (beta = -0.159, 95% CI (-0.240, -0.087)).

## Discussion and Conclusion

This study examined the relationship between general irrational beliefs, specific retirement concepts, and experiencing retirement satisfaction in individuals who had recently retired. In our correlational analysis, we confirmed that the conceptualization of retirement positively and in an active way, as a *New Start* and *Continuation,* relates to positive retirement satisfaction *(H1)*. This result coincides with previous research, confirming that retirees, who can understand their retirement as a potentially positive period, are more active, consider their life as more meaningful, and live their retirement more actively [39, 40]. We believe, that especially involvement in activities which provide positive experiences [40] can be the result of positive retirement concepts. Thus, positive retirement concepts can lead to more rewarding and socially rich lifestyles bringing more retirement satisfaction.

Our results also confirmed the opposite. Those retirees who see retirement negatively and in a passive way as an *Imposed Disruption* or *Transition to Old Age* were more dissatisfied with their retirement (*H2*). This result contributes to the previous research, which confirmed that framing retirement as a negative period is related to unfavorable retirement outcomes [39, 41]. The size of the correlation coefficients suggests that especially *Imposed Disruption* negatively contributed to retirement satisfaction. People with high scores in *Imposed Disruption* see their retirement as the loss of a highly valued activity accompanied by low retirement self-efficacy [41]. Thus, we can suppose that low retirement satisfaction in people with highly *Imposed Disruption* is an outcome of a perceived inability to establish satisfactory lifestyles after retirement.

As expected, the relationship between irrational beliefs and retirement satisfaction proved to be negative *(H3)*. It is corroborated by many previous studies, which confirmed the negative relationship between irrational beliefs and subjective well-being [29, 30, 32, 33]. Our results extend the existing knowledge by showing that the effect of irrational beliefs applies to such contextual satisfaction as satisfaction with retirement. The results thus support the plausibility of the REBT model for the retirement period and suggest that the interventions based on REBT could be effective in increasing retirement satisfaction.

This suggestion also supports specific correlations between irrational beliefs and retirement concepts. Although we found no relation between irrational beliefs and the positive retirement concepts *of New Start* and *Continuation (H4*), there was a strong middle-sized relationship between irrational beliefs and negative retirement concepts (*Imposed Disruption* and *Transition to Old Age*) (*H5)*. Although irrational beliefs are assumed to have consequences in terms of both the positive and negative side of human functioning [22, 23], our results suggest that particularly negative thinking about retirement is more associated with irrational beliefs.

The mediation analysis focused on the assumptions that retirement concepts mediate the effect of general irrational beliefs on retirement satisfaction *(H6).* The results of parallel mediation showed that general irrational beliefs predict retirement satisfaction indirectly only via the *Imposed Disruption* concept of retirement. The mediation effect was negative, which means that general irrational beliefs appear to lead to a negative view of retirement as an imposed disruptive event, and subsequently, *Imposed Disruption* intensifies retirement dissatisfaction. Hornstein and Wapner [36] characterized those who experience the retirement transition as an imposed disruption as follows: "Retirement is not experienced by these individuals as something that was *chosen.* Rather, it is felt to have been imposed from some outside source, against their own best interests. There is a feeling of abruptness about the actual ending of work and a sense of not being prepared for the changes retirement will bring." Even after entering retirement, "an underlying feeling of disquiet remains, reflecting the sense that one has made the best out of a situation that one neither sought nor desired." [[36], p. 306]. Our results suggest that general irrational beliefs can intensify such perceptions and feelings, and by this effect, a decrease in retirement satisfaction may occur. In general, irrational beliefs can intensify the feelings of retirement as an involuntary termination of employment.

Previous research showed that involuntary retirement is an extensive risk factor for retirement adjustments. Pirani, De Santis, and Zanasi [48] found that while health does not change significantly after entering retirement for those who retire formally, it worsens considerably for those who leave the labor market for another reason. Radó and Boissonneault [49] found that voluntary retirees had a higher level of subjective well-being than involuntary retirees both in the short and long term.

Considering that our participants, on average, reported satisfaction in retirement (average score slightly in the upper part of possible values), this study is best suited to address people with low retirement satisfaction, as it is the group who perceive retirement as a disruption. The findings add less to those individuals for whom retirement is a positively perceived life event. Also, those with irrational beliefs might benefit from pre-retirement intervention involving changing and mitigating general irrational beliefs and/or specific retirement-related negative concepts. In general, our results contribute to confirming the relevance of psychological resources, specifically the absence of irrational beliefs and positive retirement concepts, for retirement satisfaction.

The number of workers approaching the retirement stage of the life cycle continues to grow as life expectancy increases. Consequently, understanding the implications of retirement for individuals' well-being is of great value and a challenge for both research and policymaking. Research on well-being and life satisfaction indicates that well-being is not simply the result of adequate resources but also a predictor of future adaptation [43]. Our results showed a link between personal beliefs and retirement adjustment that might have more than just short-term effects. It may also be associated with a long-term decline in well-being and depression in retirement [10].

From our results, some implications for further research emerge. Examining the links and consequences of a negative perception of retirement seems highly justified. Some studies on this topic have recently been published [41]. The determinants of retirement perception as a disruptive and affecting event are noteworthy. Persons, who perceive an event (e.g. retirement) as undesirable or disturbing, may be more sensitive, feel greater resentment and disdain, and need more help just the same way as retirees who have still not come to terms with retirement despite having been retired for some years.

Concerning general irrational beliefs, we are of the opinion that our findings might indicate the need to develop specific content-based measures of irrational beliefs relating to retirement, not just general irrational beliefs. Gaviţa and Duţă [26] state that people may have special cognitions about themselves in different roles and positions. Specific irrational beliefs may be the key to their reactions. Assessments of irrational beliefs in particular domains of life may help subsequent treatment of problems occurring in these domains [11].

There are some limitations to this study. We used a cross-sectional research design which has its limitation for studying our research question because it does not allow disentangling whether irrational beliefs lead to lower retirement satisfaction or whether people with lower retirement satisfaction lean more often into irrational beliefs. Our model was based on the theoretical assumptions described earlier, however, a longitudinal design would be a better option for more conclusive results. We intend to tackle the problem with this approach in further research and encourage readers to do so. Research into differences between recent retirees and long-term retirees could yield more revealing results on the adjustment process and interpretation of retirement concepts. We did not include participants’ previous jobs and relationships to the job when analyzing the data in our research study. Therefore, we have no way of knowing whether the negative feelings about retirement were experienced mainly (or only) by retirees for whom work represents a key value in their life. Another limiting factor in our study may be the relatively small number of participants. Limitations also include obtaining data from an online and convenient sample of Slovak retirees, which contained higher educated participants. These limitations are balanced to some extent by focusing on discovering the deeper connections of subjective experiences within the personality. Generalizing outside the Slovak population should be cautious, as legislative and cultural variables can play a significant role in shaping irrational beliefs, retirement concepts, and retirement satisfaction. However, although the cultural context affects irrational beliefs and the perception of retirement, verification of whether the examined psychological associations have more general relevance would be beneficial. Finally, as already mentioned, a specific measure of irrational retirement beliefs could yield findings on their nature and how these relate to the variables observed. Such a measure could also indicate the potential for later counseling work aimed at modifying "irrational" personal beliefs about retirement.

## Data Availability

The datasets generated and analyzed during the current study are publicly available at https://osf.io/hrf5a/?view_only=c7eb4a604ff74d3c92d22d62569f2d32
